# The Effect of Noninvasive Telemonitoring for Chronic Heart Failure on Health Care Utilization: Systematic Review

**DOI:** 10.2196/26744

**Published:** 2021-09-29

**Authors:** Stefan L Auener, Toine E P Remers, Simone A van Dulmen, Gert P Westert, Rudolf B Kool, Patrick P T Jeurissen

**Affiliations:** 1 IQ healthcare Radboud Institute for Health Sciences Radboud University Medical Center Nijmegen Netherlands

**Keywords:** heart failure, telemonitoring, remote monitoring, health care utilization, eHealth

## Abstract

**Background:**

Chronic heart failure accounts for approximately 1%-2% of health care expenditures in most developed countries. These costs are primarily driven by hospitalizations and comorbidities. Telemonitoring has been proposed to reduce the number of hospitalizations and decrease the cost of treatment for patients with heart failure. However, the effects of telemonitoring on health care utilization remain unclear.

**Objective:**

This systematic review aims to study the effect of telemonitoring programs on health care utilization and costs in patients with chronic heart failure. We assess the effect of telemonitoring on hospitalizations, emergency department visits, length of stay, hospital days, nonemergency department visits, and health care costs.

**Methods:**

We searched PubMed, Embase, and Web of Science for randomized controlled trials and nonrandomized studies on noninvasive telemonitoring and health care utilization. We included studies published between January 2010 and August 2020. For each study, we extracted the reported data on the effect of telemonitoring on health care utilization. We used *P*<.05 and CIs not including 1.00 to determine whether the effect was statistically significant.

**Results:**

We included 16 randomized controlled trials and 13 nonrandomized studies. Inclusion criteria, population characteristics, and outcome measures differed among the included studies. Most studies showed no effect of telemonitoring on health care utilization. The number of hospitalizations was significantly reduced in 38% (9/24) of studies, whereas emergency department visits were reduced in 13% (1/8) of studies. An increase in nonemergency department visits (6/9, 67% of studies) was reported. Health care costs showed ambiguous results, with 3 studies reporting an increase in health care costs, 3 studies reporting a reduction, and 4 studies reporting no significant differences. Health care cost reductions were realized through a reduction in hospitalizations, whereas increases were caused by the high costs of the telemonitoring program or increased health care utilization.

**Conclusions:**

Most telemonitoring programs do not show clear effects on health care utilization measures, except for an increase in nonemergency outpatient department visits. This may be an unwarranted side effect rather than a prerequisite for effective telemonitoring. The consequences of telemonitoring on nonemergency outpatient visits should receive more attention from regulators, payers, and providers. This review further demonstrates the high clinical and methodological heterogeneity of telemonitoring programs. This should be taken into account in future meta-analyses aimed at identifying the effective components of telemonitoring programs.

## Introduction

### Background

Chronic heart failure (CHF) is one of the most prevalent high-cost chronic diseases affecting at least 1%-2% of the worldwide population [1]. Europe and the United States spent approximately 1%-2% of their national health care budget on this chronic disease [1,2]. Worldwide, the economic burden of CHF is estimated to be approximately US $108 billion per annum, of which US $65 billion can be attributed to direct health care costs [2]. CHF is characterized by an erratic and difficult-to-predict course. A high percentage of incurred costs are due to high readmission rates, as well as the high number of comorbidities [3].

Changes in physiological parameters such as weight, heart rate, blood pressure, and pulse oximetry may precede cardiac events. Signaling such changes through telemonitoring may enable physicians to intervene before the patient needs hospitalization or an emergency department (ED) visit [4]. Telemonitoring has been proposed as a possible strategy to tackle the challenges that CHF brings to health systems, most notably, spare use of the utilization of expensive resources. Telemonitoring has the potential to prevent hospital readmissions, thereby saving costs and improving the quality of life of these patients [5].

The technology and quality of care for patients with CHF have been evolving, which has resulted in a variety of telemonitoring programs consisting of different elements for different populations. In addition, studies on the effect of telemonitoring on health care utilization differ widely according to cointerventions, time horizons, and outcome measures. Such parameters may affect the effectiveness of telemonitoring and the comparability of studies [6]. This heterogeneity at many levels results in debates concerning the effectiveness of telemonitoring.

Studies on telemonitoring have shown mixed results with respect to health care utilization. Although some studies showed a decline, others showed no significant differences or even an increase in health care utilization. Many studies have included cointerventions within the telemonitoring intervention. Structured telephone support (STS) has often been incorporated [7-9]. As STS has been reported to reduce the number of heart failure–related hospitalizations [10], the effects found in studies that use these two interventions simultaneously may not be solely attributed to the telemonitoring program. A Cochrane study from 2015 [10] concluded that, although telemonitoring can reduce heart failure–related admissions, telemonitoring programs were not able to reduce the risk of all-cause hospitalization. A variety of systematic reviews have been performed, mostly exclusively including randomized controlled trials (RCTs). Bashi et al [11] performed an overview of systematic reviews and found that 11 of 19 systematic reviews only included RCTs. Although RCTs are considered as the gold standard for research purposes, observational studies also have merits that should not be ignored. For example, they provide a sense of the real world as opposed to experimental RCT settings [12]. Especially for diseases such as heart failure, which is known for a high degree of multimorbidity [13], RCTs may be limited by their strict inclusion and exclusion criteria. In addition, the vast majority of previous systematic reviews have only analyzed the effect of telemonitoring on hospitalizations and mortality [10,11]. Thus, limited information is available on the effect of telemonitoring programs on other outcomes, such as ED visits, length of stay, and other forms of health care utilization.

### Objective

We therefore performed a systematic review on the effect of telemonitoring for CHF on health care utilization, including both RCTs and observational studies. By including a wide variety of telemonitoring programs and outcome measures, we aim to identify the various aspects and broad impact of telemonitoring programs on the health care utilization of patients with CHF.

## Methods

### Search Strategy

PubMed, Web of Science, and Embase (Ovid) databases were searched. All authors were consulted for additional eligible studies. A detailed description of our search strategy for each database can be found in Multimedia Appendix 1. We excluded articles published before January 1, 2010 because technology and insights have rapidly evolved over the past 10 years. Our search was updated until August 4, 2020 according to the method described by Bramer [14]*.* Removal of duplications was performed according to the method described by Bramer et al [15].

### Eligibility Criteria

Telemonitoring includes a wide variety of definitions and descriptions. Generally, telemonitoring refers to the use of telecommunication to assist in the transmission of medical information and services between health care providers and patients [16]. This definition also includes STS. Many studies do not explicitly distinguish between invasive and noninvasive telemonitoring techniques. However, this review explicitly focuses on noninvasive techniques exclusively because there are significant differences between implanted monitoring devices and noninvasive telemonitoring in terms of costs and eligibility of patients [17]. Thus, we defined telemonitoring as the noninvasive application– or web-based collection and transfer of physiological data, aimed at improving quality of life or decreasing health care utilization in patients with heart failure, or both.

We included peer-reviewed studies that reported direct or indirect measurements of health care utilization such as hospitalizations, ED visits, length of stay, days of hospitalization, visits, and health care costs. We did not apply exclusion parameters concerning study design to include all available evidence. Therefore, we included RCTs, nonrandomized trials, and studies on observational data. However, we excluded studies that were not based on original data such as Markov models because these studies are based on assumptions rather than empirical data. Studies with regular telephone support initiated by nurses or health care providers (without medical indication) were excluded to focus on the effect of telemonitoring as opposed to the combined effect of telemonitoring and STS. We excluded studies from countries not belonging to the Organization for Economic Co-operation and Development to improve homogeneity in terms of the socioeconomic characteristics of the populations. Finally, studies that were not published in either English or Dutch were included. Textbox 1 presents the inclusion and exclusion criteria.

**Inclusion and exclusion criteria.**

**Inclusion criteria**

- Telemonitoring equipment within program assessed physiological parameters.
- Telemonitoring measures were shared with at least one health care provider.
- Patients are living independently and are allowed to have access to home care services but should not be admitted to a nursing home during the intervention.
- Subjects have been diagnosed with chronic heart failure.
- Outcomes included direct or indirect measures of health care utilization.
- Paper was a peer-reviewed publication.

**Exclusion criteria**

- Study was performed in countries not belonging to the Organization for Economic Co-operation and Development.
- Publication date was before January 1, 2010.
- Telemonitoring program included structured telephone support.
- Intervention program used invasive telemonitoring (eg, CardioMEMS).
- Study was not available in the English or Dutch language.
- No quantitative data with accompanied statistical analysis or measure of statistical significance was reported.
- Health care utilization measure was not reported separately (eg, combined end point with death).

### Study Selection and Data Extraction

Titles and abstracts were independently screened by 2 researchers (SLA and TEPR) for eligibility. In cases where no conclusive decision could be made, studies were included for full-text screening. Full-text screening was performed in duplicate and independently by SLA and TEPR. Disagreements were resolved by discussion until consensus was achieved or consultation with a coauthor (RBK or SAVD). Thereafter, data were extracted by one researcher (SLA) for both CHF-specific health care utilization and all-cause utilization because a reduction in CHF-specific health care utilization may not translate into an overall reduction [10]. For each study, the effect of telemonitoring on health care utilization was determined by extracting data on hospitalizations, ED visits, length of stay, days of hospitalization, visits, and health care costs. We determined the statistical significance of these results using a cut-off value of *P*<.05. If no *P* value was reported, we deemed the effect statistically significant if the reported CI did not include 1.00.

### Risk of Bias Assessment

The risk of bias assessment was performed by SLA and TEPR. For the risk of bias assessment, we used the Cochrane risk-of-bias tool for randomized trials [18] and Risk of Bias in Non-randomized Studies of Interventions tools (Cochrane) [19] for RCTs and all remaining studies, respectively. The studies were assessed in duplicate and independently. Subsequently, the individual assessments were compared for possible discrepancies. When discrepancies were observed, the studies and corresponding assessments were discussed until a consensus was reached.

## Results

### Overview

**Figure 1:**
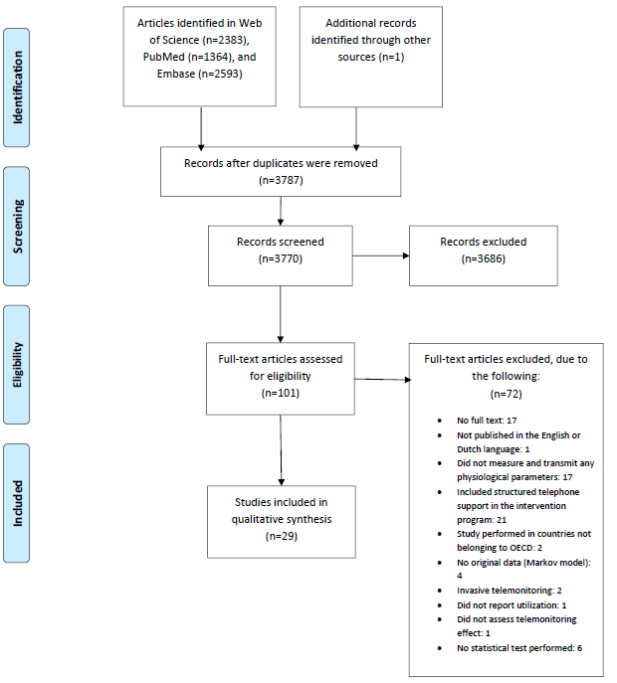
PRISMA (Preferred Reported Items for Systematic Reviews and Meta-Analyses) flowchart. OECD: Organization for Economic Co-operation and Development.

Our search strategy identified 3770 unique studies, which were then screened in the title and abstract. This resulted in 99 remaining studies. After the full-text screening, 29 studies were included. Figure 1 shows the flow diagram. The main reason for exclusion after full-text screening was because STS was a part of the intervention (19/70, 27%), followed by a lack of measuring and transmitting physiological parameters in the program (17/70, 24%). Most studies (11/29, 38%) originated in the United States.

### Characteristics of Included Studies

Table 1 presents an overview of study and program characteristics. A detailed description of the studies and their characteristics are presented in Table 2. Telemonitoring programs showed a high degree of heterogeneity in terms of physiological variables measured as shown in Multimedia Appendix 2 [4,20-47]. All studies included weight as a parameter, whereas only 4 included electrocardiography measures as a physiological parameter [20-23]. The studies used 10 unique combinations of physiological parameters. In addition, 2 studies included other measures such as lung fluid [24] and body composition [25], which were acquired by noninvasive means. Most programs (n=16) used telemonitoring in addition to usual care; regular check-up visits were still scheduled [4,22,24,26-38]. One study [20] stated that telemonitoring was used as a substitute for these visits, and 12 studies did not elaborate on this matter. The follow-up period of the studies ranged between 1 and 89 months, with most studies having a follow-up period of 6 months. Follow-up was consistent with the duration of the telemonitoring program itself, with the exception of 2 studies that also included a follow-up after the telemonitoring program was completed [24,39].

**Table 1:** Overview of the characteristics of included studies (N=29).

Study characteristics		Studies, n (%)
**Country**		
	Belgium	2 (7)
	Canada	2 (7)
	Denmark	1 (3)
	Finland	1 (3)
	Israel	2 (7)
	Italy	3 (10)
	Japan	1 (3)
	Netherlands	3 (10)
	Spain	1 (3)
	Sweden	1 (3)
	United Kingdom	1 (3)
	United States	11 (38)
**Physiological parameter assessed**		
	Weight	29 (100)
	Blood pressure	23 (79)
	Pulse oximetry	7 (24)
	Heart rate	20 (69)
	Symptom questions	14 (48)
	ECG^a^	4 (14)
**Number of patients enrolled in telemonitoring program**		
	<50	8 (28)
	50-100	9 (31)
	100-200	9 (31)
	>200	3 (10)
**Follow-up period (months)**		
	1	1 (3)
	2	1 (3)
	3	4 (14)
	6	10 (34)
	9	1 (3)
	12	8 (28)
	>12	4 (14)

^a^ECG: electrocardiography.

**Table 2:** Detailed characteristics of the included studies (N=29).

Study	Study characteristics						
	Country	Study design	Patient inclusion criteria	NYHA^a^ class	Follow-up period (months)	Intervention group (n)	Control group, n
Amir [24]	United States	Prospective	Stage C CHF^b^ with hospital admission for acute HF^c^	—^d^	6	50	N/A^e^
Bakhshi [42]	Israel	Comparison of group with telemonitoring versus group without telemonitoring	Primary diagnosis CHF index hospitalization or patients seen by emergency room, internal medicine, and cardiology specialists	—	6	44	16
Delaney [43]	United States	RCT^f^	Recently discharged from home care, eligible, and previously hospitalized for HF	III: 95 (95%)	3	50	50
Dendale [44]	Belgium	RCT	Hospitalized for fluid overload due to HF	Mean NYHA class: 3.0	6	80	80
Domingo [26]	Spain	RCT	Outpatients attended in the HF unit with NYHA classes II-IV	II: 75 (82%) and III: 17 (18%)	12	44	48
Eilat-Tsanani [45]	Israel	Before-after	NYHA classes II-IV with at least 3 admissions within a 6-month period	II:74 (52.5%); III: 55 (39%); and IV: 12 (8.5%)	12	141	N/A
Frederix [39]	Belgium	RCT	Patients participated in the TEMA-HF^g^ study by Dendale et al [44]	Mean NYHA class: 3.0	89	80	80
Hoban [27]	United States	RCT	HF diagnosis care from home health care agency	—	3	40	40
Kotooka [25]	Japan	RCT	Discharge following admission for acute HF or decompensated chronic HF	II: 142 (78.5%); III: 39 (21.5%)	31	90	91
Koulaouzidis [28]	United Kingdom	Retrospective comparison with patients that declined	Newly diagnosed (outpatient) patients with HF	II: 238 (52.5%) and III: 215 (47.5%)	12	124	329
Kraai [20]	Netherlands	Multicenter RCT	Admitted to intensive care or cardiology ward or visited outpatient HF- clinic and in need of treatment	II:39 (22%); III:100 (58.5%) and IV:33 (18.6%)	9	94	83
Lyngå [29]	Sweden	RCT	Hospitalized with NYHA classes III-IV	III: 309 (96.9%); IV: 10 (3.1%)	12	166	153
Maeng [30]	United States	Retrospective within patients	Have a diagnosis of CHF	—	70	541	N/A
Olivari [21]	Italy	RCT	Discharge from hospital after acute HF within the past 3 months	II: 163 (48.1%); III: 159 (46.9%); IV: 17 (5%)	12	229	119
Park [40]	United States	Prospective comparison between hospital and national readmission rates	Admitted for a diagnosis of acute HF	—	1	58	N/A
Pedone [31]	Italy	RCT	Diagnosis of HF and aged >64 years	II: 29 (32%); III: 51 (57%); IV: 10 (11%)	6	50	46
Riley [41]	United States	Prospective comparison with matched controls	HF admission diagnosis in the electronic health record	—	6	45	45
Seto [22]	Canada	RCT	Ambulatory patients diagnosed with HF	II: 43 (43%); II-III: 11 (11%); III: 42 (42%); IV: 4 (4%)	6	50	50
Soran [32]	United States	RCT	Diagnosis of HF in Medicare data and hospitalized for HF within 6 months	II: 183 (58.1%); III: 132 (41.9%)	6	160	155
Tompkins [33]	United States	RCT	Diagnosis of HF	—	6	193	197
Van der Burg [34]	Netherlands	Retrospective before-after study	NYHA classes III-IV	—	36	177	N/A
Veenstra [35]	Netherlands	Prospective prestudy and poststudy without controls	Discharged after admission for HF or outpatient visit and NYHA classes II-IV	II: 28 (27.5%); III: 57 (55.9%); IV: 17 (16.7%)	12	102	N/A
Vestergaard [46]	Denmark	RCT with economic analysis	Diagnosed with HF according to national guidelines and NYHA classes II-IV	Missing:13 (4.7%); II: 147 (53.6%); III: 92 (33.6%); IV: 22 (8%)	12	134	140
Villani [23]	Italy	RCT	NYHA classes III or IV during hospital stay and high risk of early rehospitalization at discharge	Mean NYHA: 3.01	12	40	40
Vuorinen [47]	Finland	RCT	Left ventricular ejection fraction<35%, NYHA ≥2, and needed regular follow-up	II: 36 (38%); III: 55 (59%); IV: 3 (3%)	6	47	47
Ware [36]	Canada	Before-after study	Diagnosed with HF and visited the HF clinic	≤II: 143 (45.4%)	6	315	N/A
White-Williams [4]	United States	Retrospective study with controls	Discharged from hospital stay and admitted to a home health care agency	—	3	29	17
Williams [37]	United States	Retrospective study with matched controls	Medicare eligible, had a diagnosis of HF, and experienced a recent (2 days) discharge from hospital	—	2	105	105
Zan [38]	United States	Prospective study with matched controls	Participants recruited from the outpatient clinic. Patients admitted to hospital program were excluded	Intervention group: I: 5 (24%); II: 9 (43%); III: 7 (33%)	3	21	20

^a^NYHA: New York Heart Association.

^b^CHF: chronic heart failure.

^c^HF: heart failure.

^d^Data not reported.

^e^N/A: not applicable.

^f^RCT: randomized controlled trial.

^g^TEMA-HF: Telemonitoring in the Management of Heart Failure.

Patient inclusion criteria for previous hospitalizations differed among the studies. If a hospital admission was required for inclusion, this was most often (n=10) within 7 days of discharge [4,23,24,29,35,37,39-42]. Inclusion was started within 1 month of index hospitalization for 2 studies [20,25], and 5 studies [21,32,43-45] included patients within 6 months. Most studies (n=12) [22,26-28,30,31,33,34,36,38,46,47] did not specify requirements regarding previous admissions for CHF or included patients from outpatient clinics. New York Heart Association (NYHA) Class II was most often reported (n=9) as the NYHA Classification with most patients [21,22,25,26,28,32,38,45,46]. NYHA Class III was reported in 8 studies as the dominant class [20,23,29,31,35,43,44,47].

### Risk of Bias Assessment

We found that most RCTs (12/16, 75%) showed at least some bias concerns, with 2 RCTs having a high risk of bias [21,27]. The main reasons for *some concerns* in the RCTs were related to the randomization process. In addition, 75% (9/12) of nonrandomized studies were assessed as having a *serious* or *critical* risk of bias. No nonrandomized studies were assessed as having a *low risk* of bias, which indicates that the strength of evidence from none of the included nonrandomized studies is equivalent to a well-performed RCT [19]. *Bias due to confounding* was the domain that most often resulted in studies being assessed with a *serious* or *critical* risk for bias. Table 3 shows the consolidated overall bias assessments. A detailed overview of the risk of bias assessment of all studies can be found in Multimedia Appendix 3 [20-23,25-27,29,31-33,39,43,44,46,47] and Multimedia Appendix 4 [4,24,28,30,34-38,40-42,45].

**Table 3:** Summary of risk of bias assessment for randomized controlled trials and nonrandomized studies.

Study		Result of bias risk assessment
**Randomized controlled trials^a^**		
	Delaney (2013) [43]	Some concerns
	Dendale (2014) [44]	Some concerns
	Domingo (2011) [26]	Some concerns
	Hoban (2013) [27]	High risk
	Kotooka (2018) [25]	Low risk
	Kraai (2016) [20]	Low risk
	Lyngå (2012) [29]	Some concerns
	Frederix (2018) [39]	Low risk
	Olivari (2018) [21]	High risk
	Pedone (2015) [31]	Some concerns
	Seto (2012) [22]	Some concerns
	Soran (2010) [32]	Some concerns
	Tompkins (2012) [33]	Some concerns
	Vestergaard (2020) [46]	Low risk
	Villani (2014) [23]	Some concerns
	Vuorinen (2014) [47]	Some concerns
**Nonrandomized studies^b^**		
	Amir (2017) [24]	Moderate risk
	Bakhshi (2011) [42]	Serious risk
	Eilat-Tsanani (2015) [45]	Critical risk
	Koulaouzidis (2019) [28]	Serious risk
	Maeng (2014) [30]	Critical risk
	Park (2019) [40]	Critical risk
	Riley (2015) [41]	Moderate risk
	Van der Burg (2020) [34]	Serious risk
	Veenstra (2015) [35]	Critical risk
	Ware (2020) [36]	Serious risk
	White-Williams (2015) [4]	Serious risk
	Williams (2016) [37]	Moderate risk
	Zan (2015) [38]	Serious risk

^a^Assessed using Cochrane risk-of-bias tool for randomized trials.

^b^Assessed using Risk of Bias in Non-randomized Studies of Interventions tool.

### Effects of Telemonitoring on Health Care Utilization

#### Overview

Telemonitoring studies used many different outcome measures, the most common of which were hospitalizations, ED visits, and health care costs. Whereas some studies report the effects of telemonitoring on specific CHF outcomes, for example, heart failure readmissions, other studies only report the effect on all-cause health care utilization. The type of analysis and effect measures differed widely among studies, with hazard ratios, odds ratios, 2-tailed *t* tests, and incidence rates being reported. Table 4 shows the (statistically significant) effects of telemonitoring on health care utilization.

**Table 4:** Effect of telemonitoring programs on health care utilization.

Study	Outcome measure															
	Hospitalization		Number of emergency department visits			Length of stay			Days of hospitalization			Visits^a^			Health care costs	
	HF^b^	AC^c^	HF	AC	HF		AC	HF		AC	HF		AC	HF		AC
Amir (2017) [24]	—^d^	+^e^	—	—	—		—	—		—	—		—	—		—
Bakhshi (2011) [42]	—	—	—	—	—		=^f^	—		—	—		—	—		—
Delaney (2013) [43]	=	+	—	—	—		—	—		—	—		—	—		—
Dendale (2014) [44]	=	=	—	—	—		—	—		=	—		—	—		=
Domingo (2011)^g^ [26]	=	—	—	—	—		—	=		—	×^h^		—	—		—
Eilat-Tsanani (2015) [45]	=	+	—	=	—		—	—		+	—		=	—		—
Frederix (2018) [39]	—	—	—	—	—		—	+		=	—		—	=		=
Hoban (2013) [27]	—	=	—	—	—		—	—		—	—		—	—		—
Kotooka (2018) [25]	=	=	—	—	—		—	—		—	—		—	—		—
Koulaouzidis (2019) [28]	=	=	—	—	—		—	+		=	—		—	—		—
Kraai (2016) [20]	=	=	—	—	—		—	—		—	+		=	—		=
Lyngå (2012) [29]	=	=	—	—	—		—	=		—	—		—	—		—
Maeng (2014) [30]	—	+	—	—	—		—	—		—	—		—	—		+
Olivari (2018)^i^ [21]	=	=	—	=	=		=	—		—	—		—	—		—
Park (2019) [40]	—	=	—	—	—		—	—		—	—		—	—		—
Pedone (2015) [31]	=	+	—	—	—		—	—		—	—		—	—		—
Riley (2015)^j^ [41]	—	=	—	—	—		—	—		=	—		—	—		—
Seto (2012) [22]	—	=	—	=	—		—	—		=	×		—	—		—
Soran (2010) [32]	—	—	—	—	—		—	—		—	—		—	—		×
Tompkins (2012) [33]	—	=	—	=	—		=	—		=	—		×	—		=
Van der Burg (2020) [34]	—	+	—	—	—		—	—		+	—		—	—		+
Veenstra (2015) [35]	+	—	—	—	—		—	—		—	—		—	—		—
Vestergaard (2020) [46]	—	—	—	—	—		—	—		—	—		—	—		+
Villani (2014) [23]	+	—	—	+	—		—	—		—	—		×	—		×
Vuorinen (2014) [47]	—	—	—	—	—		—	=		—	×		×	—		—
Ware (2020) [36]	+	+	=	=	=		=	—		—	=		—	—		—
White-Williams (2015) [4]	—	=	—	=	—		—	—		—	—		—	—		—
Williams (2016) [37]	—	=	—	—	—		—	—		—	—		×	—		×
Zan (2015) [38]	—	=	—	=	—		—	—		—	—		—	—		—

^a^Visits include all nonemergency department outpatient visits or consultations with health care provider contacts.

^b^HF: heart failure–related.

^c^AC: all-cause.

^d^Data not reported.

^e^Indicates statistically significant (*P*<.05) reduction in outcome measure.

^f^Indicates not statistically significant (*P*>.05) on outcome measure.

^g^Analysis based on the comparison between Motiva and Motiva plus.

^h^Indicates statistically significant (*P*<.05) increase in outcome measure.

^i^Analysis based on intention-to-treat analysis.

^j^Analysis based on matched-cohort analysis.

#### Hospitalization

Hospitalization was the most commonly reported outcome (24/29, 83% of studies). Out of 24 studies, 9 (38%) showed a statistically significant reduction in hospitalizations associated with telemonitoring. The remaining studies (15/24, 63%) found no effect on hospitalization. When excluding all studies with a high, critical, or serious risk of bias (n=11), 31% (4/13) of studies reported a statistically significant reduction in hospitalizations [23,24,31,43]. Although the number of studies reporting a statistically significant effect was limited, the effects found in these studies were clinically relevant. Amir et al [24] reported a temporary reduction of hospitalizations of 87% during the intervention. However, hospitalization increased by 79% when the intervention ended. Delaney et al [43] found that hospitalization rates were 19% and 38% in the intervention and control groups, respectively. Pedone et al [31] reported an incidence rate of 0.30 (95% CI 0.12-0.67), favoring the intervention group. Villani et al [23] found that the control group had almost twice the number of hospitalizations compared with the intervention group. We did not find a parameter in the study characteristics that consistently differed from the other studies that showed no effect of telemonitoring.

#### ED Visits

In total, 8 studies reported results related to the number of ED visits [4,21-23,33,36,38,45]; 7 (88%) reported no statistically significant effects of telemonitoring and 1 (13%) found a significant effect, reporting 17 ED visits in the control group (n=40) compared with 6 ED visits in the intervention group (n=40) [23].

#### Length of Stay

The outcome measure length of stay was reported in 4 studies [21,33,36,42]. None of the included studies found a statistically significant effect of telemonitoring on the length of stay.

#### Days of Hospitalization

Days of hospitalization were reported in 10 studies, of which 8 (80%) reported on all-cause days of hospitalization and 5 (50%) on days of hospitalization specifically for heart failure. There were 2 studies that showed a reduction in all-cause days of hospitalization [34,45]. The 2 studies showing a decrease in heart failure–related days of hospitalization did not result in a statistically significant decrease in all-cause days of hospitalization [28,39].

#### Visits

A total of 9 studies reported outcome measures related to non-ED health care visits. These visits comprised outpatient visits [20,22,23,26,36,45,47], primary care visits [33,45], home visits by nurse [37,45], urgent care visits [33], and telephone contacts [47]. Most (6/9, 67%) of these studies found that their telemonitoring intervention was associated with a significant increase in non-ED health care visits [22,23,26,33,37,47]. A total of 2 studies found no difference [36,45], and 1 study [20] found a decrease in non-ED visits for heart failure specifically. However, this reduction of heart failure rates did not result in a statistically significant decrease in the number of visits. Two studies were considered as having a critical [45] or serious risk of bias [36]. Both of these studies reported no effect of telemonitoring on non-ED visits.

#### Health Care Costs

A comparison of health care costs between the intervention and control groups was performed in 10 studies. Out of these 10, 3 (30%) studies showed a decrease in costs [30,34,46], 4 (40%) studies showed no statistically significant difference [20,33,39,44], and 3 (30%) studies showed a negative impact of telemonitoring on health care costs [23,32,37]. Two studies, which we assessed as having a serious or critical risk of bias, reported statistically significant decreases in health care costs [30,34]. When a decrease in costs was realized by the telemonitoring program, this was mainly due to a reduction in hospitalizations or rehospitalizations [30,34]. In addition to a reduction in hospitalizations, Vestergaard et al [46] found a reduction in outpatient care costs in the telemonitoring group. Furthermore, 3 studies finding a reduction of health care costs reported 11% [30], 35% [46], and 90% [34] reductions in health care costs. When telemonitoring was associated with an increase in costs, this was due to an increase in outpatient visits [37], an overall increase in health care utilization [32], or the costs of the telemonitoring program itself [23].

Six studies included and explicitly mentioned the costs of (the development of) the intervention in their analyses [20,23,30,32,37,46]. These studies reported large differences in the costs of the intervention, mostly due to cointerventions and specific assumptions regarding program and development costs. Multimedia Appendix 5 [20,23,30,32,37,46] provides a description of the costs. In the study by Villani et al [23], a reduction in hospitalization costs could not offset the additional costs of the intervention. This indicates a substantial influence of program costs in determining the cost-effectiveness of telemonitoring programs. An important factor for cost differences was the high cost of developing a telemonitoring program for a selected population.

## Discussion

### Principal Findings

The goal of this review was to identify the broad impact and various aspects of telemonitoring programs for patients with CHF and their effects on health care utilization. Most studies showed no statistically significant effects of telemonitoring on overall hospitalizations (14/21, 67%), ED visits (7/8, 88%), length of stay (4/4, 100%), and days of hospitalization (6/8, 75%). However, the remaining studies showed reductions in health care utilization for these measures. Overall, non-ED outpatient visits and health care costs were increased in 67% (6/9) of studies and 30% (3/10) of studies, respectively. The most ambiguously reported health care utilization measure was health care costs, which showed increases in 30% (3/10) of the studies, decreases in 30% (3/10) of the studies, and no differences in 40% (4/10) of the studies. Heart failure–specific health care utilization measures showed similar results as the overall health care utilization measures.

We found a high degree of clinical diversity among the interventions, in terms of physiological parameters as well as the targeted populations. Although most telemonitoring programs were targeted at patients in NYHA Classes II-III, a variety of additional inclusion criteria were used. In addition to clinical diversity, methodological heterogeneity was also high, as can be observed by the varying risk of bias assessments.

Some of the studies included in this review suggested that telemonitoring may result in lower hospital admission rates, but most studies did not report such reductions. This mixed effect is consistent with the current literature [10,48]. In addition, we found no clear associations between the extracted study characteristics and the effect of telemonitoring and a reduction in hospitalizations. However, we found that 67% (6/9) of studies reported an increase in non-ED outpatient visits when telemonitoring was used.

Thus far, the effect of telemonitoring on non-ED visits has received little attention. However, the early warning designs of telemonitoring might also attract additional use of frontline services and false-positive alarms. An overview of systematic reviews [11] showed that 2 of 19 systematic reviews included non-ED visits in their outcomes [49,50]. These systematic reviews included 5 studies, 1 [51] of which can be classified as telemonitoring based on our definition. This RCT showed a substantial increase in non-ED visits and phone calls for telemonitoring compared with usual care. Our results suggest that this may be the case for most telemonitoring programs, as we found that 67% (6/9) of studies showed an increase in non-ED visits. One study found a decrease in heart failure–related non-ED visits. This study [20] explicitly stated that “patients allocated to the intervention group were only allowed to visit the cardiologist or HF-nurse in case of an absolute need for intervention.” In addition, 83% (5/6) of studies, which found an increase in visits, had telemonitoring as an additional component in addition to usual care [22,26,37,47], and 17% (1/6) did not state this explicitly [33]. This stresses the need to treat telemonitoring as a substitute for regular care, rather than an addition to regular care, that is, if a reduction in health care utilization is the primary aim. Five studies reported an increase in non-ED visits and hospitalizations as outcome measures. Villani et al [23] found an increase in non-ED visits simultaneously with a reduction in hospitalizations. The other 4 studies [22,26,33,37] reported an increase in non-ED visits and found no effect on hospitalization. This indicates that additional visits can be a side effect of telemonitoring rather than a prerequisite for effective telemonitoring programs in terms of reduced health care utilization. Therefore, telemonitoring programs may become more cost-effective if they pay more attention to decreasing these visits.

The effect of telemonitoring on health care costs has been inconsistent across studies. Health care costs were severely affected by the costs of telemonitoring programs, which showed large differences. These differences were attributable to both assumptions regarding the development costs per patient and the actual cost differences. These cost differences can have a detrimental effect on the cost-effectiveness and financial viability of the program. No studies included indirect cost savings or expenses from a patient perspective, such as a reduction or increase in travel costs.

### Strength and Limitations

A major strength of our study is the inclusion of various study designs and outcome measures for health care utilization. Therefore, this review offers a broader scope than other reviews [11]. The inherent weakness of including nonrandomized studies was the introduction of biases because of case mixes. This was demonstrated by the results of the Risk of Bias in Non-randomized Studies of Interventions tool, which showed a high degree of bias in the domain of baseline confounding. The exclusion of studies also using STS within their telemedicine program resulted in the exclusion of a significant number of studies. However, the exclusion of these studies also increases the relevance of our study for those interested in the stand-alone effect of telemonitoring on health care utilization.

The main limitation of this review is the lack of a meta-analysis and thus a limited ability to draw strong conclusions regarding the effect of telemonitoring on health care utilization. However, this review clearly shows why meta-analyses are difficult to perform on this subject and, if performed, should be interpreted with caution. We observed high heterogeneity within the study populations, telemonitoring programs, and outcomes. Therefore, a meta-analysis was not appropriate for this study. In addition, the results of meta-analyses of other studies may only be applicable to small subsets of populations, interventions, and outcomes and thus may not represent the true effect of telemonitoring on health care utilization because of limited external validity.

### Implications for Practice

This study has several practical implications. The finding that telemonitoring often increases non-ED visits has consequences for the workload of outpatient clinics. These additional non-ED visits may occur because of a variety of reasons such as false positives, true positives, equipment malfunction, and whether telemonitoring is used as additional or substitute care. Detecting true positives is the primary aim, as it prevents more expensive health care utilization and improves the quality of life. Although false-positive alerts and equipment malfunctions may be reduced by improving technology and algorithms [52], our results suggest that addition instead of substitution is likely to remain, resulting in additional non-ED visits. Health care providers must be aware of this increase and adopt organizational structure of the outpatient clinic or find other ways to mitigate this increase, such as using telemonitoring as a substitute rather than additional care [20] or outsourcing technical difficulties experienced by patients to medical service centers [34].

This review showed that telemonitoring might shift health care utilization to outpatient settings, as opposed to only reducing inpatient admissions. This may complicate adoption, as the benefits may not be attributable to the same stakeholder as the costs. This is especially the case for health care systems where outpatient care is delivered by organizations other than inpatient care, such as that of Germany. In such cases, conflicting interests are to be expected, and health care payers have to come to an agreement with health care providers to overcome these issues [53]. Health care regulators can facilitate this process by creating and supporting new payment models such as shared savings and lump-sum payment models.

Finally, the high costs of developing telemonitoring programs for a selected population can diminish and even cancel future monetary gains of reduced health care consumption [23]. One way of suppressing the costs of telemonitoring can be achieved through the use and development on a larger scale. Villani et al [23] showed that developing a telemonitoring system for 40 patients was not financially viable. In contrast, Vestergaard et al [46] reduced the costs of the program per patient by targeting a larger population, namely a whole region of North Denmark constituting 6700 patients. Health care providers should either use the scale realized by a third party or codevelop telemonitoring systems with other health care providers to realize possible scale advantages. In addition, in general, substitutive programs are expected to achieve higher financial savings.

### Future Research

Future studies should consider clinical diversity by including subgroup meta-analyses or performing meta-regression, as opposed to pooled meta-analyses [54]. As mentioned previously, we found a high degree of clinical and methodological diversity [54]. Despite this, some meta-analyses [48,55] have found a low to moderate effect for certain health care utilization measures. The presence of high clinical diversity and low statistical heterogeneity may be due to minimal marginal effects of components in the telemonitoring program; a part of the intervention did not affect the outcome measure. If this is the case, certain (combinations of) telemonitoring components do not add value to the intervention. Yun et al [56] and Kotb et al [57] performed such analyses on all-cause mortality and hospitalization in patients with CHF. Certain program characteristics, such as having 3 or more physiological parameters [56] or including an electrocardiograph [57], were statistically associated with a reduction in all-cause mortality and hospitalization, respectively. Similarly, essential and effective parts of telemonitoring for reducing other health care utilization can be identified, thereby supporting the development of (cost-) effective telemonitoring programs. Clear descriptions of the intervention and context are needed to perform such analyses. This review can be used as guidance for forming subgroups or variables of interest for meta-regressions. As there are many more factors that may affect the impact of telemonitoring on health care utilization, qualitative research may be used to develop hypotheses and guide meta-regression protocols.

### Conclusions

This review investigated the effects of telemonitoring programs on different aspects of health care utilization. Telemonitoring has the potential to reduce hospitalization rates. However, this was not achieved in most studies, as the number of non-ED visits increased in the majority of studies. The effect of telemonitoring on health care costs is highly ambiguous and depends on the effectiveness of the intervention in reducing health care utilization as well as on the costs of the telemonitoring program itself. Health care providers and payers should be aware that the majority of current telemonitoring programs do not result in a reduction in health care utilization and may even increase health care utilization by increasing the number of non-ED visits. Possible payer strategies should be focused at increasing the scale to reduce program costs and implement telemonitoring as a substitute to reduce possible increases in outpatient visits. Nevertheless, more focus is needed to determine the essential factors of telemonitoring programs that reduce health care utilization.

## Appendix

Multimedia Appendix 1 Search queries for PubMed, Web of Science, and Embase.

Multimedia Appendix 2 Detailed characteristics of telemonitoring programs.

Multimedia Appendix 3 Risk of bias assessment: Cochrane Risk of Bias tool for randomized trials.

Multimedia Appendix 4 Risk of bias assessment: Risk of Bias in Non-randomized Studies of Interventions tool for non-randomized studies.

Multimedia Appendix 5 Costs of telemonitoring programs.

## Abbreviations

CHF: chronic heart failure
ED: emergency department
NYHA: New York Heart Association
RCT: randomized controlled trial
STS: structured telephone support
