# Supplementary material for: The Effect of Noninvasive Telemonitoring for Chronic Heart Failure on Health Care Utilization: Systematic Review
Source: J Med Internet Res. 2021 Sep 29;23(9):e26744. doi: 10.2196/26744 (PMC8515232; doi:10.2196/26744)
Supplement: Multimedia Appendix 1 [file jmir_v23i9e26744_app1.pdf]

Multimedia appendix : Search queries for Pubmed, Web of Science and Embase

Database	Components	Search query
Pubmed	Telemonitoring	Mobile applications[MeSH] OR "Telemedicine"[Mesh:NoExp] OR Telemetry[MeSH] OR Wireless Technology[Mesh] OR App[tiab] OR Apps[tiab] OR eHealth [tiab] OR e-health[tiab] OR Electronic Application*[tiab] OR mHealth[tiab] OR m-health[tiab] OR Mobile Application*[tiab] OR Mobile Health [tiab] OR Remote monitoring [tiab] OR Remote Sens*[tiab] OR Software Application*[tiab] OR Telehealth[tiab] OR Telemetr*[tiab] OR Telemonitoring[tiab] OR Wireless Technolog*[tiab] OR Tele-monitoring[tiab] OR Tele-health[tiab]
	Heart failure	Heart Failure[MesH] OR Cardiac decompensation[tiab] OR Cardiac Failure*[tiab] OR cardial decompensation[tiab] OR Cardial failure*[tiab] OR decompensatio cordis[tiab] OR Heart Decompensation[tiab] OR Heart failure*[tiab] OR myocardial decompensation[tiab] OR Myocardial Failure*[tiab]
	Filters	Language=English or Dutch and date of publication >=2010
Web of Science (SCI-EXPANDED,SSCI, A&HCI, ESCI)	Telemonitoring	App OR Apps OR eHealth OR e-health OR Electronic Application* OR mHealth OR m-health OR Mobile Application* OR Mobile Health OR Remote monitoring OR Remote Sens* OR Software Application* OR Telehealth

		OR Telemetr* OR Telemonitoring OR Wireless Technolog*
	Heart failure	Cardiac decompensation OR Cardiac Failure* OR cardial decompensation OR Cardial failure* OR decompensatio cordis OR Heart Decompensation OR Heart failure* OR myocardial decompensation OR Myocardial Failure*
	Filters	DOCUMENT TYPES: (ARTICLE OR REVIEW OR EARLY ACCESS) AND PUBLICATION YEARS: (2020 OR 2019 OR 2018 OR 2017 OR 2016 OR 2015 OR 2014 OR 2013 OR 2012 OR 2011 OR 2010
Embase (Ovid)	Telemonitoring	(exp telehealth/ OR exp telemetry/ OR exp mobile application/ OR wireless communication/) OR<br>(App OR Apps OR eHealth OR e-health OR Electronic Application* OR mHealth OR m-health OR Mobile Application* OR Mobile Health OR Remote monitoring OR Remote Sens* OR Software Application* OR Telehealth OR Telemetr* OR Telemonitoring OR Wireless Technolog*).ti,ab,kw.
	Heart failure	exp heart failure/ OR (Cardiac decompensation OR Cardiac Failure* OR cardial decompensation OR Cardial failure* OR decompensatio cordis OR Heart Decompensation OR Heart failure* OR myocardial decompensation OR Myocardial Failure*).ti,ab,kw.

	Filters	Year>=2010 AND Article Type= Review OR Article
