# Supplementary material for: The Effect of Noninvasive Telemonitoring for Chronic Heart Failure on Health Care Utilization: Systematic Review
Source: J Med Internet Res. 2021 Sep 29;23(9):e26744. doi: 10.2196/26744 (PMC8515232; doi:10.2196/26744)
Supplement: Multimedia Appendix 2 [file jmir_v23i9e26744_app2.pdf]

Reference	Telemonitoring program characteristics					
	Weight	Blood pressure	Pulse oximetry	Heart rate	Symptom questions	ECG[a]
Amir [24]	Y[b]	N[c]	N	N	N	N
Bakhshi [42]	Y	N	N	N	N	N
Delaney [43]	Y	Y	Y	Y	Y	N
Dendale [44]	Y	Y	N	Y	N	N
Domingo [26]	Y	Y	N	Y	Y	N
Eilat-Tsanani [45]	Y	N	N	N	N	N
Frederix [39]	Y	Y	N	Y	N	N
Hoban [27]	Y	Y	N	Y	N	N
Kotooka [25]	Y	Y	N	Y	N	N
Koulaouzidis [28]	Y	Y	N	Y	N	N
Kraai [20]	Y	Y	N	Y	Y	Y
Lyngå [29]	Y	N	N	N	N	N
Maeng [30]	Y	N	N	N	Y	N
Olivari [21]	Y	Y	Y	Y	N	Y
Park [40]	Y	Y	N	N	Y	N
Pedone [31]	Y	Y	Y	Y	N	N
Riley [41]	Y	Y	Y	Y	N	N
Seto [22]	Y	Y	N	N	Y	Y
Soran [32]	Y	N	N	N	Y	N

Tompkins [33]	Y	Y	Y	Y	Y	N
Van der Burg [34]	Y	Y	N	Y	U	N
Veenstra [35]	Y	Y	N	Y	Y	N
Vestergaard [46]	Y	Y	N	Y	Y	N
Villani [23]	Y	Y	N	Y	N	Y
Vuorinen [47]	Y	Y	N	Y	Y	N
Ware [36]	Y	Y	N	Y	Y	N
White-Williams [4]	Y	Y	Y	Y	Y	N
Williams [37]	Y	Y	Y	Y	N	N
Zan [38]	Y	Y	N	Y	N	N

[a]ECG: electrocardiography.

[b]Y: Yes, parameter was assessed.

[c]N: No, parameter was not assessed.
