# Supplementary material for: The Effect of Noninvasive Telemonitoring for Chronic Heart Failure on Health Care Utilization: Systematic Review
Source: J Med Internet Res. 2021 Sep 29;23(9):e26744. doi: 10.2196/26744 (PMC8515232; doi:10.2196/26744)
Supplement: Multimedia Appendix 3 [file jmir_v23i9e26744_app3.pdf]

	Randomization process	Deviations from intended interventions	Missing outcome data	Measurement of the outcome	Selection of the reported result	Overall
Delaney, 2013	?	+	+	+	?	!
Dendale, 2014	?	+	+	+	?	!
Domingo, 2011	?	+	?	+	?	!
Hoban, 2013	?	?	+	+	?	–
Kotooka, 2018	+	+	+	+	+	+
Kraai, 2016	+	+	+	+	+	+
Lynga, 2012	?	+	+	+	+	!
Frederix, 2018	+	+	+	+	+	+
Olivari,2018	?	–	+	+	+	–
Pedone, 2015	?	+	+	+	+	!
Seto,2012	+	?	?	+	+	!
Soran, 2010	?	+	+	+	?	!
Tompkins, 2012	?	?	+	+	?	!
Villani, 2014	+	+	+	+	?	!
Vuorinen, 2014	?	+	+	+	?	!
Vestergaard, 2020	+	+	+	+	+	+

+ Low risk
? Some concerns
– High risk
