# Supplementary material for: The Effect of Noninvasive Telemonitoring for Chronic Heart Failure on Health Care Utilization: Systematic Review
Source: J Med Internet Res. 2021 Sep 29;23(9):e26744. doi: 10.2196/26744 (PMC8515232; doi:10.2196/26744)
Supplement: Multimedia Appendix 4 [file jmir_v23i9e26744_app4.pdf]

Author, year	Baseline confounding	Selection bias	Classification of intervention	Deviation from intended intervention	Missing data	Measurement of outccomes	Selection of reported results	Overall risk of bias
Amir, 2017	Moderate	Low	Low	Low	Moderate	Low	Moderate	Moderate
Bakhshi, 2011	Serious	Low	Low	Low	No information	Moderate	Serious	Serious
Eilat-Tsanani, 2015	Critical	Critical	Low	Low	Serious	Low	Moderate	Critical
Koulaouzidis, 2019	Serious	Low	Low	Low	Low	Low	Moderate	Serious
Maeng, 2014	Critical	Critical	Low	Serious	Critical	Low	Moderate	Critical
Park,2019	Critical	Low	Low	Low	Low	Low	Moderate	Critical
Riley, 2015	Moderate	Low	Low	Low	Low	Low	Moderate	Moderate
Van der Burg, 2020	Serious	Low	Low	Low	Low	Low	Moderate	Serious
Veenstra, 2015	Critical	Low	Low	Low	Low	Low	Moderate	Critical
Ware, 2020	Serious	Low	Low	Low	Low	Low	Moderate	Serious
White-Williams, 2015	Serious	Low	Low	Moderate	Low	Low	Moderate	Serious
Williams, 2016	Moderate	Low	Low	Low	Low	Low	Moderate	Moderate
Zan, 2015	Serious	Low	Low	Low	Low	Low	Moderate	Serious
