# Supplementary material for: The Effect of Noninvasive Telemonitoring for Chronic Heart Failure on Health Care Utilization: Systematic Review
Source: J Med Internet Res. 2021 Sep 29;23(9):e26744. doi: 10.2196/26744 (PMC8515232; doi:10.2196/26744)
Supplement: Multimedia Appendix 5 [file jmir_v23i9e26744_app5.pdf]

Author, year	Description of telemonitoring costs	Costs
Kraai, 2016	The cost of the intervention was defined as the costs of the ICT-guided-DMS with CDSS, the costs of the telemonitoring devices and handling of the alarms (personnel) and were calculated as a fixed price over the follow-up period.	€1729 per patient per 9 months
Maeng, 2014	The cost was determined on a per member per months basis for each member for the number of the months during which the member was enrolled in the program.<br>The cost associated with case management activities for the members participating in this program was not separately identified	Healthcare utilization differed $216 in favor of the intervention group, which resulted in a saving of 3.3 dollar per 1.0 dollar invested in the telemonitoring program.
Soran, 2011	Used data on the 6-month cost of the program from the company that delivered the telemonitoring system	$804 per patient per 6 months
Vestergaard, 2020	Costs of the telehealthcare solution, excluding costs of monitoring. Software development and education were divided among the expected number of CHF patients in the region (6700 patients). Basic operation costs and running development of apps were divided among the expected number of HF and COPD patients in the region (10500 patients)	£218,05 per patient per 12 months
Villani, 2014	Composed of development of the system, pre-programmed laboratory and instrumental tests, and personnel costs.	Incremental costs of €154,360 for 40 patients per 12 months.
Williams, 2016	The remote monitoring intervention program cost includes:<br>equipment, monitoring, maintenance, marketing, and additional personnel costs	$300 per patient per 12 months
